# Supplementary material for: DMRforPairs: identifying Differentially Methylated Regions between unique samples using array based methylation profiles
Source: BMC Bioinformatics. 2014 May 15;15:141. doi: 10.1186/1471-2105-15-141 (PMC4046028; doi:10.1186/1471-2105-15-141)
Supplement: Additional file 2 — DMRforPairs output for the comparison of A431-MCF7 and NA17018-NA17105. Please start from the HTML files in each folder. Available via the BMC Bioinformatics website. [file 1471-2105-15-141-S2.zip › 1394847754114233_MOESM2_ESM/A431_MCF7/figures/19301.pdf]

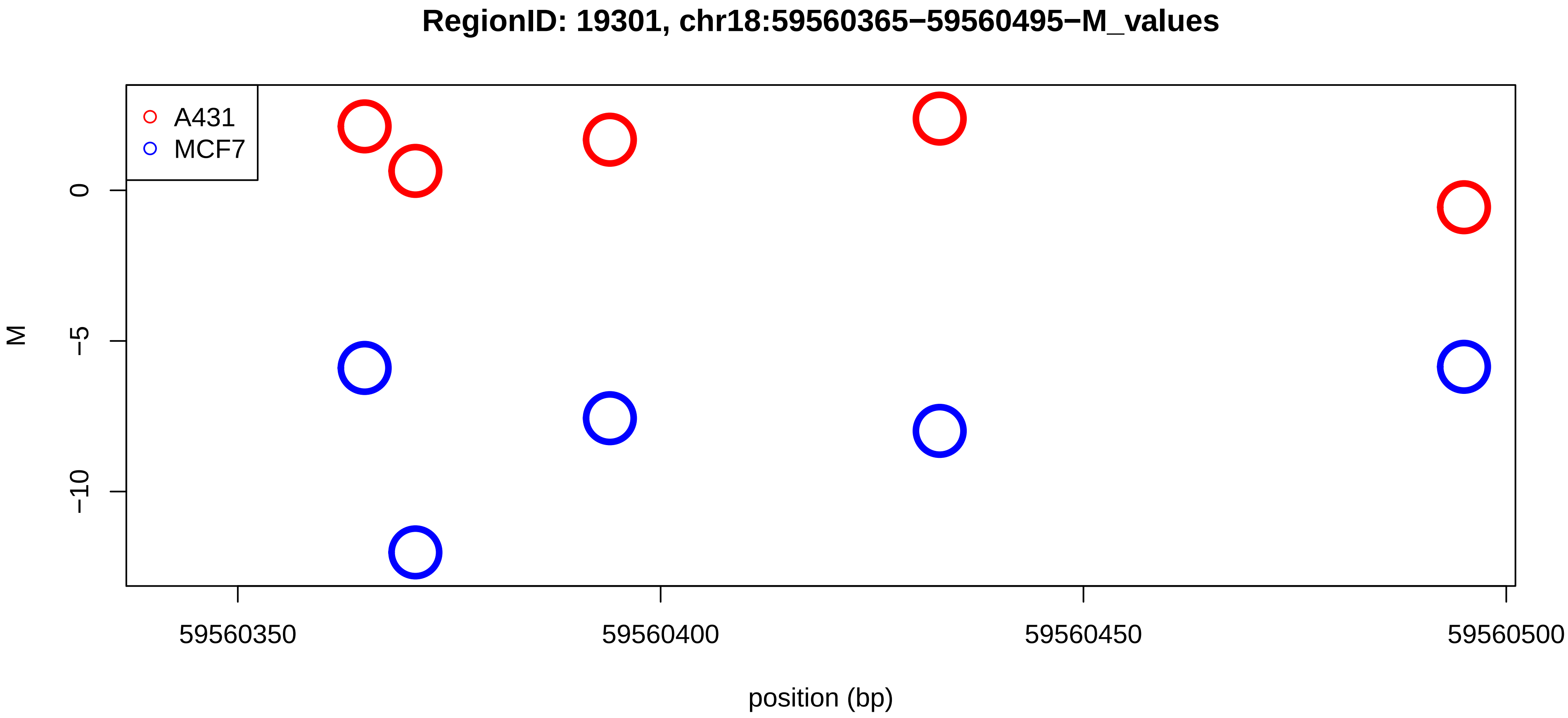
RegionID: 19301, chr18:59560365−59560495−M_values
A431
MCF7
M
0
−5
−10
59560350
59560400
59560450
59560500
position (bp)

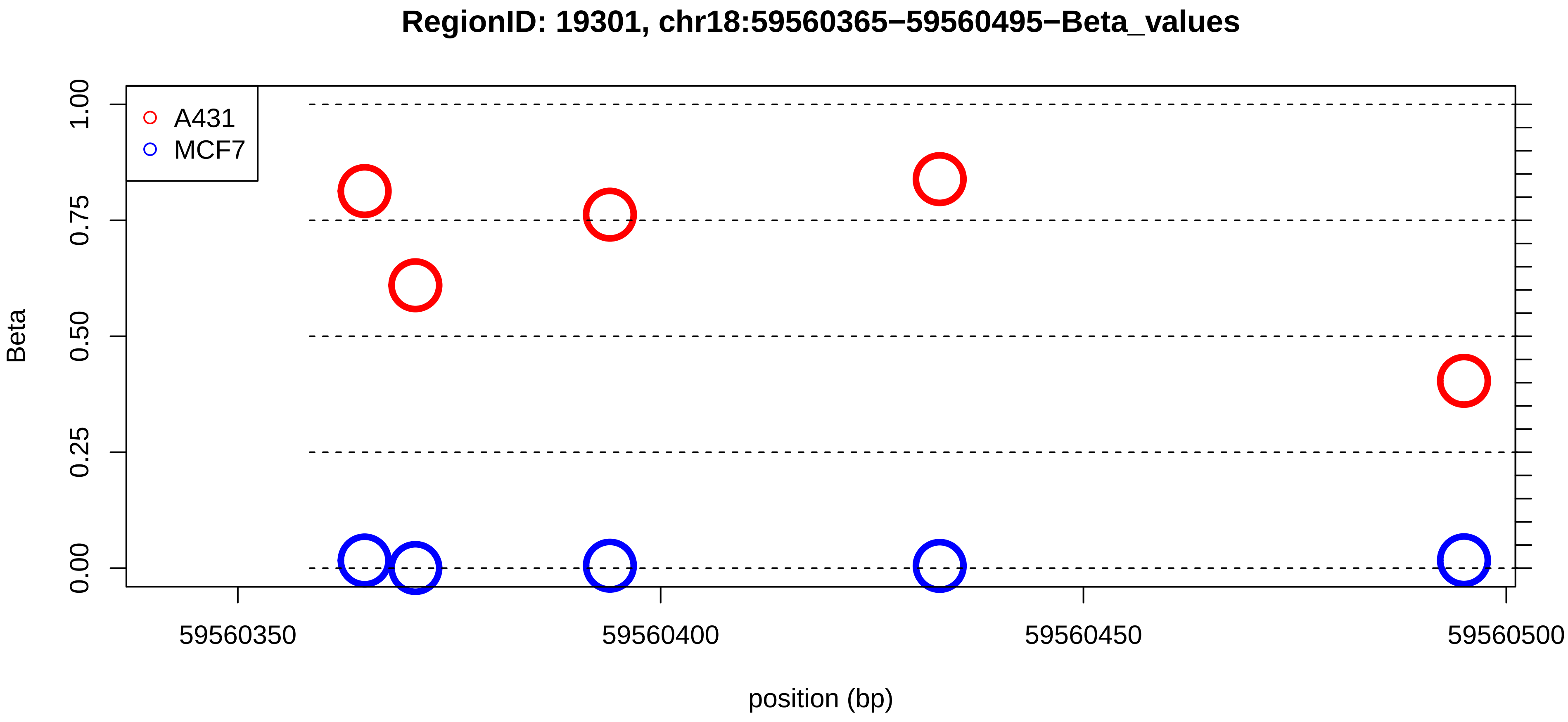
RegionID: 19301, chr18:59560365−59560495−Beta_values
A431
MCF7
Beta
0.00
0.25
0.50
0.75
1.00
59560350
59560400
59560450
59560500
position (bp)

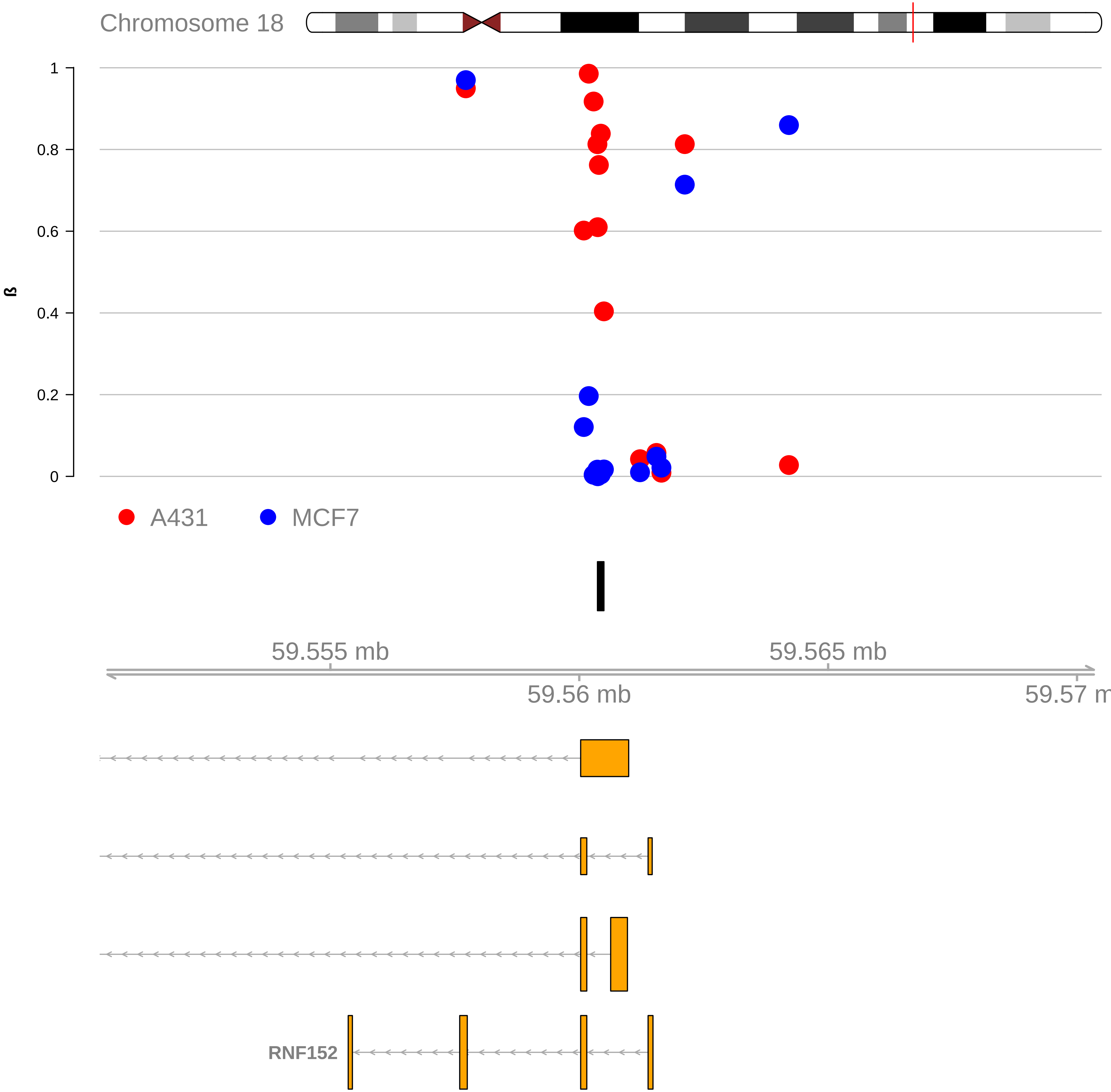
Chromosome 18
1
0.8
0.6
0.4
0.2
0
ß
A431
MCF7
59.555 mb
59.565 mb
59.56 mb
59.57 m
RNF152
